# Injuries in Physical Education Teacher Students: Differences between Sex, Curriculum Year, Setting, and Sports

**DOI:** 10.1155/2023/8643402

**Published:** 2023-01-05

**Authors:** Maarten Barendrecht, Carl C. Barten, Willem van Mechelen, Evert Verhagen, Bouwien C. M. Smits-Engelsman

**Affiliations:** ^1^Amsterdam Collaboration on Health and Safety in Sports, Department of Public and Occupational Health, Amsterdam Movement Sciences & Amsterdam Public Health Institute, Amsterdam UMC (location VUmc), Amsterdam, Netherlands; ^2^Avans+ Improving Professionals, Claudius Prinsenlaan 140, Breda, Netherlands; ^3^Haagsche Hogeschool, Academie voor Sportstudies, Mr. P. Droogleever Fortuynweg 22, Den Haag, Netherlands; ^4^Sportgeneeskunde Rotterdam, Jan Leentvaarlaan 37-47, Rotterdam, Netherlands; ^5^Division of Exercise Science and Sports Medicine (ESSM), Department of Human Biology, Faculty of Health Sciences, University of Cape Town, Cape Town, South Africa; ^6^School of Human Movement and Nutrition Sciences, Faculty of Health and Behavioural Sciences, University of Queensland, Brisbane, Australia; ^7^School of Public Health, Physiotherapy and Population Sciences, University College Dublin, Dublin, Ireland; ^8^Center of Human Movement Sciences, University Medical Center Groningen, Groningen, Netherlands; ^9^University of Cape Town, Faculty of Health Sciences, Department of Health and Rehabilitation Sciences, Old Main Building, Groote Schuur Hospital, Cape Town, South Africa; ^10^Physical Activity Sport and Recreation, Faculty Health Sciences, North West University, Potchefstroom 2520, South Africa

## Abstract

**Background:**

Injury risk is high in Physical Education Teacher Education (PETE) students. Insights into specific injury locations per sex, setting, sports, and curriculum year are needed to develop preventive measures.

**Purpose:**

To compare injury distributions by body locations in PETE students and how these distributions differ by sex, type, onset, curriculum year, settings, or sports.

**Methods:**

In a historical cohort study over 14 years, data from 2899 students (male 76.2%, *n* = 1947) enlisted in the first three years of a PETE curriculum were analysed. Injuries reported at the institution's medical facility were categorised per sex, body location, onset, type, setting, sports, and curriculum year.

**Results:**

Forty-three percent (*n* = 1247) of all students (female 54.9%, *n* = 523, male 37.2%, and *n* = 429) reported a total of 2129 injuries (freshmen 56.4%; 2^nd^ year 28.2%; 3^rd^ year 15.5%). The most prevalent sudden onset injury locations (63.4% of all injuries) were the ankle (32.5%) and knee (16.6). The most prevalent gradual onset injuries were the lower leg (27.8%) and knee (25.2%). Joint/ligament injuries (45.8%) and muscle/tendon injuries (23.4%) were the most prevalent injury types. Proportions for injury locations and injury types differed significantly between curriculum years. Injury prevalence per setting and sport differed significantly between the sexes. Injury locations differed significantly between sports and between the sexes per sport.

**Conclusion:**

A differential approach per injury location, onset, type, sex, setting, sports, and curriculum year is needed to develop adequate preventive measures in PETE studies. The engagement of precurricular, intracurricular, and extracurricular stakeholders is needed in the development of these measures.

## 1. Introduction

Students in education for sports-related professions are subjected to high cumulative sports loads over a prolonged period. Consequently, they are at high risk of sustaining an injury during their study period [1–6]. In the short term, sports injuries negatively impact sports performance [7]. In the long term, injuries can lead to arthritis, decreased levels of physical activity and quality of life, and work disability [8–10]. In the Netherlands, physical education teacher education (PETE) students participate in more than 250 hours of intracurricular (i.e., part of the PETE curriculum) sports classes per year during the first three years of their curriculum. Most PETE students also participate in extracurricular sports (i.e., leisure time or competitive sports outside the PETE curriculum) as well (262 min/wk on average) [2]. Although in a recent study in Flemish freshman PETE students, a multifactorial preventive program showed promising results in some injury categories, the overall effect was nonsignificant [11]. A better understanding of the relationship between risk groups or activities and their injuries could help develop targeted injury prevention measures. Intracurricular sports with high injury risks are track and field athletics, gymnastics, and team ball sports [2, 6, 12]. Most extracurricular injuries occur in soccer [2, 6]. A recent study over the first 3 years of the PETE curriculum shows that injury risk is highest during the freshman year and that female students have significantly higher injury odds for overall and intracurricular injuries [12]. Knowledge of the prevalence of injury locations, types, and onset, as well as the differences between sexes, curriculum years, and sports types is needed. Specific risk factors for the most frequently reported injuries and sports types could then be investigated in this population. Based on these risk factors and (sport-specific) injury mechanisms, targeted prevention strategies can be developed or applied for specific injuries and sports [13]. Recent studies on injuries in PETE students have focused on (semesters in) the freshman year only [1, 2, 4, 5, 14]. Injury locations and sports types from this period might not represent the whole curriculum. Therefore, an investigation of all three curriculum years (the fourth year consists mainly of internships) is needed to provide full insight into the prevalence of injuries in PETE students. Furthermore, studies on single cohorts with relatively low injury counts cannot differentiate injury distributions between subgroups. For this study, data on the prevalence of sports injuries by body locations, injury types, onset by sex, curriculum year, setting, and sports types were analysed over 14 years. The primary question was “Which body locations and injury characteristics are most prevalent in sports injuries of male and female PETE students during the first three years of the PETE curriculum?” The secondary question was “How do distributions of injury locations and characteristics during the PETE study differ between sex, curriculum years, settings, or sports types?”

## 2. Methods

In a historical cohort study [15], all new injuries reported at the medical facility of The Hague School for Sports Studies (HSSS) between August 2000 and June 2014 were analysed. Data from injury or student records were used anonymously with permission from the institution and following European privacy legislation [16].

### 2.1. Subjects

All students enlisted in physical education teacher education at the HSSS between August 2000 and June 2014 were included in this study. Since the fourth year of the PETE curriculum consists mainly of internships, fourth-year students were excluded from the analyses.

### 2.2. Injury Definition and Registration

The definition of injury for this study was “Any new musculoskeletal complaint related to sports participation for which medical advice was sought at the medical facility of the HSSS.” This complies with the medical attention injury definition for multisports events [17]. At the medical facility of the HSSS, voluntary free medical consultation was provided twice a week. Injuries were diagnosed by the same sports physical therapist and sports physician over the complete period of 14 years. Advice was given on treatment options or restrictions regarding sports participation. Students were obliged to report injuries at this facility to be excused from (active) participation in sports classes or exams if necessary. Data from student and injury records were collected as previously described [12]. In short, from all new musculoskeletal complaints reported at the medical facility, injury location, type, onset, sports, setting, and curriculum year were collected. During the study period, no specific injury prevention program was introduced.

### 2.3. Data Analysis

For each student included in the study, their age at the start of the curriculum (in years) and sex were recorded. For students who sustained more than one new injury, all injuries were used for analysis. All injury data from the first three curriculum years were recoded to injury locations, types, mode of onset (sudden or gradual), and time loss categories as proposed by Fuller et al. [18]. The reported setting (intracurricular or extracurricular) and sports activity were categorised. Incomplete injury records were used only if they could be traced to an individual student. For these records, missing data were categorised as unknown.

Demographic data of PETE students were calculated in mean and standard deviation (sd) for age (in years), as well as frequencies and percentages for sex. The mean ages were compared by sex using the independent *T*-test. For injuries, frequencies and percentages for time loss (yes, no, unknown) and duration of symptoms before consultation (<1 week, 1 week–4 weeks, >4 weeks, and unknown) were calculated as previously described [12] and compared by sex using *χ*^2^ analyses. Overall and subgroup frequencies and percentages for injuries were reported with 95% confidence intervals (95% CI). Proportions were compared for body locations, injury types and onset (sudden/gradual) by sex, curriculum year, setting (intracurricular or extracurricular), and sports using *χ*^2^ analyses or the Fisher–Freeman–Halton exact test. For all analyses, *α* was set at 0.05. Statistical Package for the Social Sciences (SPSS) version 27 (IBM, Chicago) was used for all analyses.

## 3. Results

Over the full study period, 2899 PETE students (male 76.2% and *n* = 1947) with a mean age at enlistment of 19.3 years (sd 2.1) were at one time enlisted at the HSSS. Male students (19.6 and sd 2.2), on average, were almost a year older (0.93, 95% CI 0.79–1.07, and *p* < 0.05) than female students (18.7 and sd 1.5). Overall, 43.0% (95% CI 41.4%–44.8%) of all students (*n* = 1247) reported at least one injury (1–7 injuries). For female students, this percentage (54.9% and 95% CI 51.8%–58.1%; *n* = 523) was significantly higher (*p* < 0.05) than for male students (*n* = 724, 37.2% and 95% CI 35.0%–39.3%). A total number of 2129 injuries (male *n* = 1142, 53.6%) was reported. Overall, 1290 injuries (60.6% and 95% CI 58.5%–62.7%) were time loss injuries (male *n* = 701, 61.4% and 95% CI 58.6%–64.2%; female *n* = 589, 59.7% and 95% CI 56.6%–62.7%). A flow diagram of baseline demographics of included students and registered injuries per curriculum year can be found in the supplementary file here: baseline demographics of included students and amounts of registered injuries per curriculum year. Percentages for time loss and duration of symptoms before the consultation were not significantly different for female students compared to male students.

### 3.1. Injury Locations by Sex and Onset

Table 1 shows injury prevalence per sex and curriculum year. Comparisons of main groupings (head/neck, trunk, upper limb, and lower limb) for injury locations showed that lower limb injuries (*n* = 1462) comprised 67.0% (95% CI 65.0%–69.0%) and upper limb injuries (*n* = 434) comprised 20.4% (95% CI 18.7%–22.1%) of all injuries. The distribution of injuries by main groupings was not significantly different between male and female students.

The most frequently reported injury locations in male students were the ankle (*n* = 264, 23.1% and 95% CI 20.7%–25.5%), the knee (*n* = 220, 19.3%, and 95% CI 17.0%–21.6%), the shoulder/clavicula (*n* = 130, 11.4% and 95% CI 9.6%–13.2%), and the lower leg (*n* = 118, 10.3% and 95% CI 8.5%–12.1%). The back/pelvis/sacrum, the foot/toe, and the thigh also showed prevalences above 5%.

The most frequently reported injury locations in female students were the ankle (*n* = 202, 20.5% and 95% CI 18.0%–23.0%), the knee (*n* = 197, 20.0% and 95% CI 17.5%–22.5%), the lower leg (*n* = 114, 11.6%, and 95% CI 9.6%–13.6%), the back/pelvis/sacrum (*n* = 94, 9.5%, and 95% CI 7.7%–11.3%), the shoulder/clavicula (*n* = 84, 8.5% and 95% CI 6.8%–10.2%), and the thigh (*n* = 57, 5.8% and 95% CI 4.3%–7.3%) (Table 1). Distributions for these frequencies were significantly different between male and female students (*p* < 0.05). For no other injury location, the prevalence for either male or female students was higher than 4.7%.

**Figure 1:**
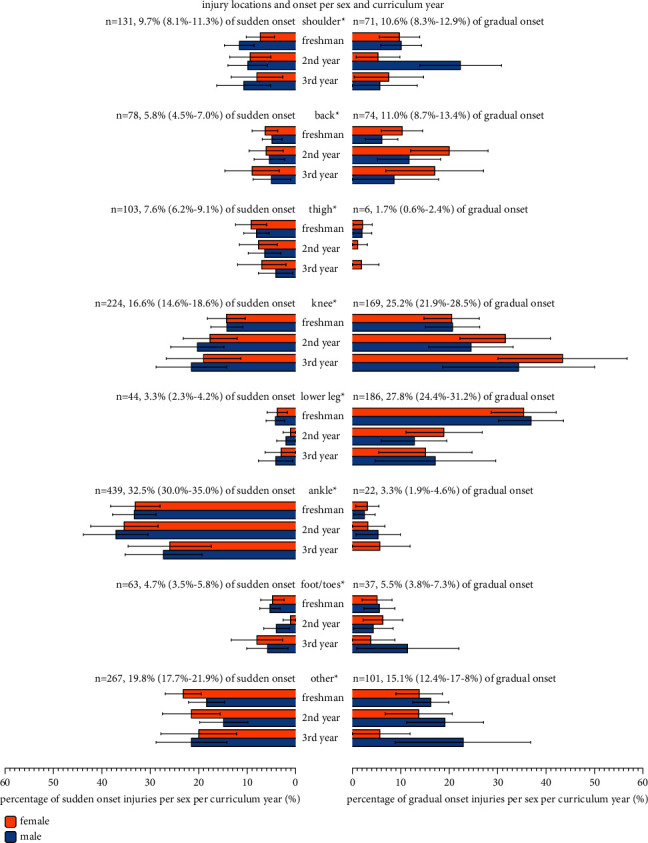
Prevalence of injury locations by onset in percentage (and 95% confidence intervals) of all injuries per sex per curriculum year. ^*∗*^For 110 injuries, the onset was unknown.

Sudden onset injuries (*n* = 1349, 63.4%, and 95% CI 61.3%–65.4%) were more frequently reported than gradual onset injuries (*n* = 670, 31.5% and 95% CI 28.8%–34.2%). Male students (*n* = 753, 65.9% and 95% CI 63.9%–68.0%) reported a significantly (*p* = 0.01) higher percentage of sudden onset injuries compared to female students (*n* = 596, 60.4% and 95% CI 58.3%–62.5%). For sudden onset injuries, the ankle (*n* = 439, 32.5% and 95% CI 30.0%–35.0%), knee (*n* = 224, 16.6% and 95% CI 14.6%–18.6%), shoulder/clavicula (*n* = 131, 9.7% and 95% CI 8.1%–11.3%), and thigh (*n* = 103, 7.6% and 95% CI 6.2%–9.1%) were the most frequently reported injury locations (Figure 1). For gradual onset injuries, the lower leg (*n* = 186, 27.8% and 95% CI 24.4%–31.2%), knee (*n* = 169, 25.2% and 95% CI 21.9%–28.5%), back/pelvis/sacrum (*n* = 74, 11.0% and 95% CI 8.7%–13.4%), and shoulder/clavicula (*n* = 71, 10.6% and 95% CI 8.3%–12.9%) were most frequently reported. Distributions per sex for the most common injury locations were for either onset not significantly different.

### 3.2. Injury Locations by the Injury Type

Overall, 976 (45.8% and 95% CI 43.7%–48.0%) injuries were diagnosed as joint and ligament injuries. Muscle and tendon injuries (*n* = 498 and 23.4% 95% CI 21.6%–25.2%) and fractures and bone stress injuries (*n* = 260, 12.1%, and 95% CI 10.8%–13.8%) were frequently diagnosed as well (supplementary file 2: injury prevalence per injury location by injury types and sex). No significant differences in injury types were found between male and female students. Most ankle injuries (*n* = 411, 88.2%, and 95% CI 85.3%–91.1%) were sprain/ligament injuries. Knee injuries were more diverse with 143 (34.3% and 95% CI 29.7%–38.8%) sprain/ligament injuries, 118 (28.3% and 95% CI 24.0%–32.0%) lesions of meniscus or cartilage (including patellofemoral pain syndrome) and 60 (14.4% and 95% CI 11.0%–17.8%) of the tendon. Shoulder/clavicula injuries were most commonly diagnosed as muscle/tendon injuries (*n* = 87, 40.7% and 95% CI 34.1%–47.4%), dislocation/subluxations (*n* = 38, 17.8% and 95% CI 12.6%–22.9%) or sprain/ligament injuries (*n* = 31, 14.5% and 95% CI 9.8%–19.2%). Lower leg injuries were predominantly (*n* = 169, 72.8% and 95% CI 67.1%–78.6%) diagnosed as other bone injuries (medial tibial stress syndrome) and as muscle ruptures/tears/strains/cramps (*n* = 43, 18.5% and 95% CI 13.5%–23.5%). Most back/pelvis sacrum injuries were diagnosed as joint/ligament injuries (n = 72, 43.6% and 95% CI 36.1%–51.2%), muscle ruptures/tears/stains/cramps (n = 35, 21.2% and 95% CI 15.0%–27.4%). For another 45 injuries, the injury type was unknown (27.3% and 95% CI 20.5%–34.1%). Thigh injuries were predominantly muscle ruptures/tears/strains/cramps (*n* = 100, 87.0% and 95% CI 80.8%–93.1%), and foot/toe injury types were diverse.

### 3.3. Injury Locations, Onset, and Types by Curriculum Year

Of all registered injuries, 56.4% were freshman injuries, 28.2% were 2^nd^ year injuries, and 15.5% were 3^rd^ year injuries (Figure 1). Distributions per sex were not significantly different. Comparisons of proportions of injury locations by curriculum year (Table 1) showed significant differences (*p* < 0.05). Ankle and knee injuries were most prevalent in all three curriculum years. The proportion of lower leg injuries sharply decreased after the freshman year. This decrease was predominantly caused by a decrease in gradual onset lower leg injuries (freshman year 36.1% and 95% CI 30.1%–43.6%; 2^nd^ year 15.9% and 95% CI 10.7%–21.1%; 3^rd^ year 15.9% and 95% CI 8.3%–29.6%) compared to knee injuries (freshman year 20.6% and 95% CI 16.6%–24.6%; 2^nd^ year 28.0% and 95% CI 21.6%–34.4%; 3^rd^ year 39.9% and 95% CI 29.5%–50.0%) and most other gradual onset injuries. In sudden onset injuries, the differences between curriculum years were less obvious (Figure 1).

Comparison between curriculum years for injury types showed significant differences compatible with those found in gradual onset injuries. The contribution of fractures and bone stress injuries (predominantly lower leg injuries) was more than twice as high during the freshman year (15.7% and 95% CI 13.6–17.7%) compared to the 2^nd^ and 3^rd^ year (7.3%, 95% CI 5.2%–9.4% and 8.5%, 95% CI 5.5%–11.5%, respectively). In contrast, joint and ligament injuries increased from 41.9% (95% CI 39.1%–44.7%) in the freshman year to 51.2% (95% CI 47.2%–55.2%) and 50.5% (95% CI 45.1%–55.9%) in the 2^nd^ and 3^rd^ years.

### 3.4. Injury Locations by Setting and Sports Type

**Figure 2:**
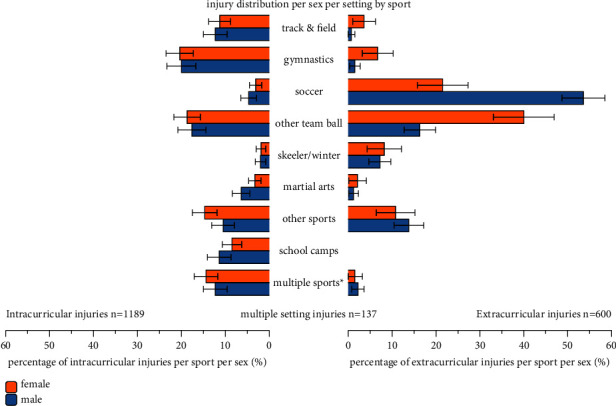
Prevalence of injuries per sport as percentage (and 95% confidence intervals) of all injuries per sex per setting. ^*∗*^Another 137 injuries were sustained during activities in multiple settings.

In male students, 48.2% (95% CI 45.4%–51.1%) of all injuries occurred in intracurricular sports activities, and 35.5% (95% CI 32.7%–38.2%) occurred in extracurricular activities (Table 2). These percentages were significantly different in female students, with 64.6% (95% CI 61.7%–67.7%) and 19.8% (95% CI 17.3%–22.2%), respectively (*p* < 0.05). For most individual injury locations, the pattern was similar. Still, in both sexes, higher percentages of lower leg injuries were reported intracurricular, whereas higher percentages of knee injuries were reported extracurricular. Injury distribution by sports type (Figure 2) differed significantly (*p* < 0.05) between male and female students. Male students sustained most injuries during soccer (22.6% and 95% CI 20.2%–25.0%), other team sports (14.4% and 95% CI 12.4%–16.5%), multiple sports (12.3% and 95% CI 10.4%–14.3%), and gymnastics (10.2% and 95% CI 8.5%–12.0%). Female students sustained most injuries during other team sports (20.8% and 95% CI 18.2%–23.3%), multiple sports (16.3% and 95% CI 14.0%–18.6%), gymnastics (14.9% and 95% CI 12.7%–17.1%), and track and field athletics (8.0% and 95% CI 6.3%–9.7%). Soccer injuries predominantly occurred during extracurricular activities (80.4% and 95% CI 76.1%–84.8%), whereas 58.4% (95% CI 53.4%–63.4%) of other team sports injuries occurred intracurricular. More than 88% of gymnastic and track and field athletics injuries occurred in male and female students during intracurricular activities.

**Figure 3:**
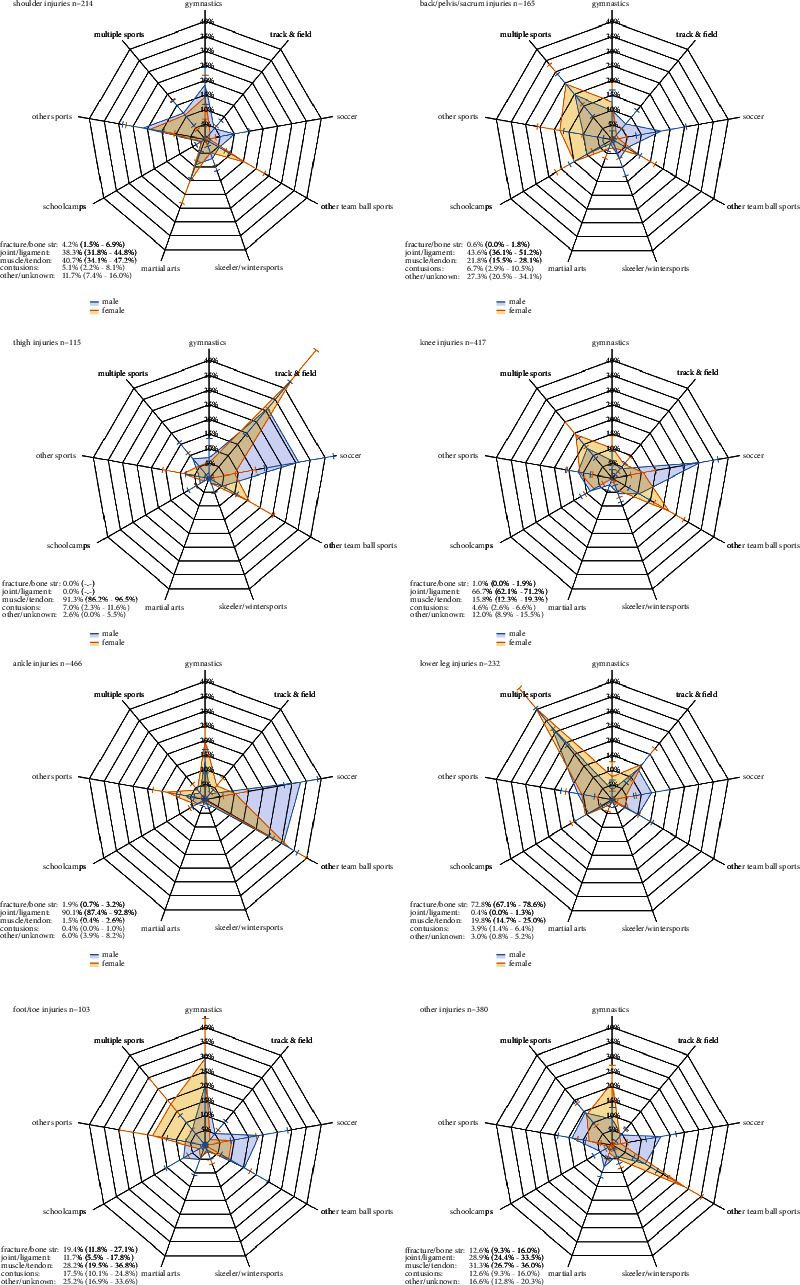
Injury distribution (and 95% confidence intervals) per injury location and sex by sports (bone str. = bone stress).

Figure 3 shows analysis of most common injury locations by sport that showed significant differences between injury locations overall and per sex. In all injury locations, the proportion of soccer injuries was higher in male students, whereas this was higher in female students for other team sports. Apart from that, ankle injuries also occurred frequently in gymnastics and thigh injuries occurred predominantly in track and field. Most lower leg injuries occurred in multiple sports. The other injury locations showed more diverse distributions but with specific patterns by sex and sports.

## 4. Discussion

This study aimed to identify the most prevalent injury locations and characteristics in PETE students and to compare their distributions between sexes, curriculum years, settings, and sports types. Our study showed that over the first three curriculum years, both in male and female Dutch PETE students, injuries to the knee, ankle, lower leg, shoulder, and back/pelvis were most prevalent. Joint and ligament injuries were most prevalent in both sexes, followed by muscle and tendon injuries, fractures, and bone stress injuries. Whereas knee and ankle injuries were most prevalent over all curriculum years, the proportion of (gradual onset) lower leg injuries decreased after the freshman year. Most injuries occurred in soccer (predominantly male extracurricular), other team ball sports (both intracurricular and extracurricular), intracurricular gymnastics, and track and field. Overall and location-specific injury distributions per sport were significantly different between the sexes.

The results of our study shed new light on injury characteristics in PETE students over their full curriculum. Recent studies on injury characteristics in PETE students investigated freshman students only [1, 2, 4, 5]. Our study's distribution of injury locations is compatible with earlier studies but showed a higher proportion of knee and ankle injuries during the freshman year and overall, especially compared to lower leg injuries [1, 2, 4, 6]. The majority of these lower leg injuries were gradual onset injuries. In our registration of medical attention injuries, possibly only more persistent lower leg injuries were registered, compared to other studies with prospective registration of all injuries [1, 2, 4, 6]. Intercultural differences in student fitness or curriculum load might also be of influence. In Flemish PETE studies, the curriculum starts with 12 weeks of sports classes, whereas in Dutch PETE studies, periods of 6 or 7 weeks of sports classes are followed by a 3-week or 4-week period without practical classes. Although a serious problem in freshman students [8, 16], the percentage of gradual onset lower leg injuries in our study dropped during the 2^nd^ and 3^rd^ years. These results are compatible with the decrease of new lower leg injuries after the first trimester, found in freshman students [14]. Whether these injuries have dissolved in later years, students learn to cope with them, or they do not report them anymore, needs further investigation. The increase in the proportion of gradual onset knee injuries over the years in our study indicates that more prolonged/sustained preventive measures over all curriculum years are needed for these injuries. This holds for acute knee and ankle injuries.

Comparison between sexes showed a significant but relatively small difference in distribution by injury location. However, female athletes sustained a significantly larger part of their injuries (64.6%) during intracurricular activities than male students (48.2%). This is in line with the higher overall intracurricular injury risk we found for females compared to male PETE students (rate ratio female to male: 2.38, 95% CI 1.97 to 2.87) who completed their full curriculum [19]. In Flemish freshman PETE students, a comparable but nonsignificant higher intracurricular injury risk was found for female students, whereas in extracurricular injuries, it was the other way around [4]. A possible bias for the higher extracurricular prevalence we found in male students is a larger cumulative exposure time to extracurricular sports activities in male students [4]. Furthermore, the attribution of injuries to a certain setting or sport differs between studies. In one study, unsupervised training in the context of curricular sports is categorised as an extracurricular activity [4]. Like in the study of Twellaar et al. [6], we included both sudden and gradual onset injuries in our distribution between settings and sports because this classification was closest to the original injury registration and best represented the students' perceptions of the onset of their injuries. According to the students, more than 50% of injuries attributed to multiple sports were associated with intracurricular sports. How these perceptions must be interpreted needs further investigation. In extracurricular sports, students compete at their own level of play and training. In many intracurricular sports that are new to students, extensive repetitions of new sports techniques might lead to gradual onset injuries. On top of that, the differences in physical capacities between sexes might explain the higher percentage of intracurricular injuries in female students compared to male students [20].

A comparison of injuries by sports types showed that team ball sports were most frequently (32.5%) reported. The higher contribution of (predominantly extracurricular) soccer (22.6%) in male students compared to female (6.5%) students could be explained by the popularity of male soccer in the Netherlands [21]. Other team ball sports were more frequently reported in female (20.8%) compared to male (14.4%) students, but for both sexes, most of these injuries (58% and 59%, respectively), were sustained during intracurricular sports activities. Gymnastic and track and field athletics injuries were also predominantly (more than 88%) sustained during intracurricular activities. These results align with contemporary results in Dutch freshman PETE students [2].

The found distributions by sex and sports implicate that for soccer injuries (male students) and other extracurricular team sports (both sexes), risk factors should be derived from sports representative of extracurricular activities. For intracurricular team sports, gymnastics, and track and field athletics, risk factors should be investigated in intracurricular activities. Their risk factors and injury mechanisms might be more related to mistakes in technique and high repetitive loads due to new motor skills that have to be acquired in sports unfamiliar to PETE students. The higher injury rates for practice sessions than for sports classes found in earlier studies confirm this assumption [6, 11]. In first-year students, one third of all injuries are gradual onset noncontact injuries, and during curricular practices, 44% of injury mechanisms are noncontact [22]. To develop tailor-made injury prevention programs for PETE students, the distribution of these mechanisms for specific injury locations and sports needs further investigation.

Goossens et al. found a nonsignificant (*p*=0.061) 20% reduction in injury rates after implementing a combined programme of injury awareness and preventive strategies in their regular PETE programme. Their program showed a significant reduction in extracurricular injury risk but not in intracurricular injury risk [23]. Considering the higher proportion of intracurricular injuries than extracurricular injuries, enhanced prevention strategies are needed. Compared to extracurricular injuries, a more differentiated approach for intracurricular activities might help reduce the risk of intracurricular injury. The specific injury profiles per injury location, setting, and sports per sex found in this study could help specify preventive measures further. For most ankle injuries, wearing an ankle brace could reduce the risk of injury by more than half [24]. Both for knee and ankle injuries, neuromuscular training has shown to be effective in team sports players [24, 25]. To reduce the number of thigh injuries, besides strength training interventions [26], load management of fatigued students in track and field and team sports is warranted [27]. Considering the already high load on PETE students and related high injury risk in the first year of their curriculum [12], extrinsic risk factors such as rapid increases in training load [28] also need to be considered in gradual onset injuries in general and specifically of the lower leg and knee. A precurricular neuromuscular training program for novice PETE students could address (sex specific) risks factors for specific injuries. Such training programs can improve landing kinematics related to both sudden and gradual onset knee injuries in female athletes [29, 30]. The addition of basic exercises to get familiar with new techniques in gymnastics and other (new) intracurricular sports could further help novice (female) students to adapt to new physical loads before the curriculum starts. Reducing the overall physical load of the curriculum for female (freshmen) students could help minimize the risk difference between sexes for all intracurricular sports, irrespective of specific injury locations.

The strength of our study was the prospective registration of injuries over all curriculum years over 14 years by the same sports medical professionals. The resulting high number of registered injuries enabled subanalyses that helped get more insight into the injuries in PETE students. The interpretation of the results of our study also needs to consider the weaker points. The registration of injuries at the HSSS was initiated before the publication of the consensus statement of Fuller et al. [18], and injury locations and categories were categorised retrospectively. According to the student, the registered sport/setting during which an injury occurred is not always the sport/setting responsible for that injury. In sudden and gradual onset injuries, the accumulative load from earlier activities can contribute to an injury event [31]. Our study was conducted in a single institution on Dutch PETE students, but our results align with those from other studies on Dutch PETE students [1, 2]. However, interpretation of our results in other countries should consider intercultural differences in sports participation and curriculum load [2–5].

### 4.1. Perspective

Compared to previous studies on freshman students [1, 2, 4, 5], our analyses of 2129 injuries from three curriculum years allow for enhanced recommendations for research on risk factors and preventive measures in PETE students [11, 23]. For intracurricular sports that students are unfamiliar with, novice athletes' risk factors and injury mechanisms should be targeted. A precurricular preventive program aimed at these sports could help (female) students adapt to the high curricular load before the freshman year. Including Nordic hamstring exercises in precurricular preventive programs could reduce the high number of thigh injuries in sprint-related sports [26]. For these thigh injuries and for (knee and lower leg) gradual onset injuries, the effect of load management and distribution over the curriculum years needs investigation. The higher injury risk for female students [12, 19], irrespective of injury location, could further be targeted with a reduction of their physical load compared to male students. The high contribution of (extracurricular) team sports to acute ankle and knee injuries implies the need to investigate existing sports-specific preventive programs in PETE students [11, 24, 25]. These programs should be implemented in extracurricular sports, adjusted to students' individual extracurricular playing levels. The preventive effect of ankle braces during intracurricular and extracurricular team sports and gymnastics should be investigated [24]. To tackle the diverse nature and distribution of injuries over the curriculum in PETE students, a differentiated multisetting approach including precurricular, intracurricular, and extracurricular stakeholders is needed.

## Figures and Tables

**Table 1:** Injury prevalence and proportions per injury location per sex and curriculum year for all injuries. Proportions (and 95% confidence intervals) are calculated as percentage of total injuries per sex per curriculum year. For 37 injuries, the exact injury location was unknown.

Body locations	Sex	Freshmen			2nd year			3rd year			Total		
		*n*	%	95% CI (%)	*n*	%	95% CI (%)	*n*	%	95% CI (%)	*n*	%	95% CI (%)
Head and neck total	Male	15	2.3	1.2–3.4	5	1.6	0.2–3.0	4	2.4	0.1–4.7	24	2.1	1.3–2.9
	Female	10	1.9	0.7–3.1	9	3.2	1.2–5.2	6	3.7	0.8–6.6	25	2.5	1.5–3.5
	Total	25	2.1	1.3–2.9	14	2.3	1.1–3.5	10	3.0	1.2–4.8	49	2.3	1.7–2.9

Upper limbs total	Male	129	19.5	16.5–22.5	84	26.7	21.8–31.6	30	18.2	12.3–24.1	243	21.3	18.9–23.7
	Female	114	21.2	17.7–24.7	51	17.9	13.4–22.4	26	15.9	10.3–21.5	191	19.4	16.9–21.9
	Total	243	20.3	18.0–22.6	135	22.5	19.2–25.8	56	17.0	12.9–21.1	434	20.4	18.7–22.1

Shoulder/clavicula	Male	70	10.6	8.3–12.9	45	14.3	10.4–18.2	15	9.1	4.7–13.5	130	11.4	9.6–13.2
	Female	47	8.7	6.3–11.1	23	8.1	4.9–11.3	14	8.5	4.2–12.8	84	8.5	6.8–10.2
	Total	112	9.8	8.1–11.5	68	11.3	8.8–13.8	29	8.8	5.7–11.9	214	10.1	8.8–11.4

Upper arm	Male	1	0.2	0.0–0.5	1	0.3	0.0–0.9	0	0.0	0.0–0.0	2	0.2	0.0–0.5
	Female	1	0.2	0.0–0.6	0	0.0	0.0–0.0	1	0.6	0.0–1.8	2	0.2	0.0–0.5
	Total	2	0.2	0.0–0.5	1	0.2	0.0–0.6	1	0.3	0.0–0.9	4	0.2	0.0–0.4

Elbow	Male	10	1.5	0.6–2.4	5	1.6	0.2–3.0	3	1.8	0.0–3.8	18	1.6	0.9–2.3
	Female	7	1.3	0.3–2.3	10	3.5	1.4–5.6	2	1.2	0.0–2.9	19	1.9	1.0–2.8
	Total	17	1.4	0.7–2.1	15	2.5	1.3–3.7	5	1.5	0.2–2.8	37	1.7	1.2–2.2

Forearm	Male	3	0.5	0.0–1.0	2	0.6	0.0–1.5	1	0.6	0.0–1.8	6	0.5	0.1–0.9
	Female	3	0.6	0.0–1.3	3	1.1	0.0–2.3	0	0.0	0.0–0.0	6	0.6	0.1–1.1
	Total	6	0.5	0.1–0.9	5	0.8	0.1–1.5	1	0.3	0.0–0.9	12	0.6	0.3–0.9

Wrist	Male	22	3.3	1.9–4.7	15	4.8	2.4–7.2	6	3.6	0.8–6.4	43	3.8	2.7–4.9
	Female	28	5.2	3.3–7.1	3	1.1	0.0–2.3	2	1.2	0.0–2.9	33	3.3	2.2–4.4
	Total	50	4.2	3.1–5.3	18	3.0	1.6–4.4	8	2.4	0.7–4.1	76	3.6	2.8–4.4

Hand/finger/thumb	Male	23	3.5	2.1–4.9	16	5.1	2.7–7.5	5	3.0	0.4–5.6	44	3.9	2.8–5.0
	Female	27	5.0	3.2–6.8	12	4.2	1.9–6.5	7	4.3	1.2–7.4	46	4.7	3.4–6.0
	Total	50	4.2	3.1–5.3	28	4.7	3.0–6.4	12	3.6	1.6–5.6	90	4.2	3.3–5.1

Trunk total	Male	44	6.6	4.7–8.5	27	8.6	5.5–11.7	13	7.9	3.8–12.0	84	7.4	5.9–8.9
	Female	45	8.4	6.1–10.7	33	11.6	7.9–15.3	21	12.8	7.7–17.9	99	10.0	8.1–11.9
	Total	89	7.4	5.9–8.9	60	10.0	7.6–12.4	34	10.3	7.0–13.6	183	8.6	7.4–9.8

Sternum/ribs/abdomen	Male	8	1.2	0.4–2.0	2	0.6	0.0–1.5	3	1.8	0.0–3.8	13	1.1	0.5–1.7
	Female	2	0.4	0.0–0.9	2	0.7	0.0–1.7	1	0.6	0.0–1.8	5	0.5	0.1–0.9
	Total	10	0.8	0.3–1.3	4	0.7	0.0–1.4	4	1.2	0.0–2.4	18	0.8	0.4–1.2

Back (upper and lower)/pelvis/sacrum	Male	36	5.4	3.7–7.1	25	7.9	4.9–10.9	10	6.1	2.4–9.8	71	6.2	4.8–7.6
	Female	43	8.0	5.7–10.3	31	10.9	7.3–14.5	20	12.2	7.2–17.2	94	9.5	7.711.3
	Total	79	6.6	5.2–8.0	56	9.3	7.0–11.6	30	9.1	6.0–12.2	165	7.8	6.7–8.9

Lower limbs total	Male	459	69.3	65.8–72.8	197	62.5	57.2–67.8	115	69.7	62.7–76.7	771	67.5	64.8–70.2
	Female	360	66.9	62.9–70.9	189	66.3	60.8–71.8	106	64.6	57.3–71.9	655	66.4	63.5–69.3
	Total	819	68.3	65.7–70.9	386	64.3	60.5–68.1	221	67.2	62.1–72.3	1426	67.0	65.0–69.0

Hip/groin	Male	24	3.6	2.2–5.0	6	1.9	0.4–3.4	6	3.6	0.8–6.4	36	3.2	2.2–4.2
	Female	16	3.0	1.6–4.4	10	3.5	1.4–5.6	0	0.0	0.0–0.0	26	2.6	1.6–3.6
	Total	40	3.3	2.3–4.3	16	2.7	1.4–4.0	6	1.8	0.4–3.2	62	2.9	2.2–3.6

Thigh	Male	40	6.0	4.2–7.8	13	4.1	1.9–6.3	5	3.0	0.4–5.6	58	5.1	3.8–6.4
	Female	33	6.1	4.1–8.1	15	5.3	2.7–7.9	9	5.5	2.0–9.0	57	5.8	4.3–7.3
	Total	73	6.1	4.7–7.5	28	4.7	3.0–6.4	14	4.3	2.1–6.5	115	5.4	4.4–6.4

Knee	Male	108	16.3	13.5–19.1	69	21.9	17.3–26.5	43	26.1	19.4–32.8	220	19.3	17.0–21.6
	Female	91	16.9	13.7–20.1	63	22.1	17.3–26.9	43	26.2	19.5–32.9	197	20.0	17.5–22.5
	Total	199	16.6	14.5–18.7	132	22.0	18.7–25.3	86	26.1	21.4–30.8	417	19.6	17.9–21.3

Lower leg	Male	91	13.7	11.1–16.3	16	5.1	2.7–7.5	11	6.7	2.9–10.5	118	10.3	8.5–12.1
	Female	81	15.1	12.1–18.1	20	7.0	4.0–10.0	13	7.9	3.8–12.0	114	11.6	9.6–13.6
	Total	172	14.3	12.3–16.3	36	6.0	4.1–7.9	24	7.3	4.5–10.1	232	10.9	9.6–12.2

Achilles tendon	Male	8	1.2	0.4–2.0	1	0.3	0.0–0.9	3	1.8	0.0–3.8	12	1.1	0.5–1.7
	Female	7	1.3	0.3–2.3	3	1.1	0.0–2.3	2	1.2	0.0–2.9	12	1.2	0.5–1.9
	Total	15	1.3	0.7–1.9	4	0.7	0.0–1.4	5	1.5	0.2–2.8	24	1.1	0.7–1.5

Ankle	Male	150	22.7	19.5–25.9	80	25.4	20.6–30.2	34	20.6	14.4–26.8	264	23.1	20.7–25.5
	Female	104	19.3	16.0–22.6	69	24.2	19.2–29.2	29	17.7	11.9–23.5	202	20.5	18.0–23.0
	Total	254	21.2	18.9–23.5	149	24.8	21.3–28.3	63	19.1	14.9–23.3	466	21.9	20.1–23.7

Foot/toe	Male	36	5.4	3.7–7.1	12	3.8	1.7–5.9	11	6.7	2.9–10.5	59	5.2	3.9–6.5
	Female	26	4.8	3.0–6.6	8	2.8	0.9–4.7	10	6.1	2.4–9.8	44	4.5	3.2–5.8
	Total	62	5.2	3.9–6.5	20	3.3	1.9–4.7	21	6.4	3.8–9.0	103	4.8	3.9–5.7

Total	Male	662	100		315	100		165	100		1142	100	
	Female	538	100		285	100		164	100		987	100	
	Total	1200	100		600	100		329	100		2129	100	

**Table 2:** Prevalence of injury locations and 95% confidence intervals (95% CI) for intracurricular and extracurricular injuries and for injuries sustained in multiple settings. ^*∗*^For 35 injuries, no exact body location was registered. ^*∗∗*^For 69 injuries, the setting was unknown.

Body locations		Intracurricular			Extracurricular			Multiple settings			Total^*∗∗*^	
		*n*	%	95% CI (%)	*n*	%	95% CI (%)	n	%	95% CI (%)	*n*	%
Shoulder/clavicula	Male	70	53.8	45.3–62.4	43	33.1	25.0–41.2	7	5.4	1.5–9.3	130	100
	Female	50	59.5	49.0–70.0	16	19.0	10.7–27.4	6	7.1	1.6–12.7	84	100
	Total	120	56.1	49.4–62.7	59	27.6	21.6–33.6	13	6.1	2.9–9.3	214	100

Back/pelvis/sacrum	Male	31	43.7	32.1–55.2	19	26.8	54.4–81.9	11	15.5	7.1–23.9	71	100
	Female	64	68.1	58.7–77.5	13	13.8	6.9–20.8	13	13.8	6.9–20.8	94	100
	Total	95	57.6	50.0–65.1	32	19.4	13.4–25.4	24	14.5	9.2–19.9	165	100

Thigh	Male	29	50.0	37.1–62.9	21	36.2	23.8–48.6	5	8.6%	1.4–15.8	58	100
	Female	41	71.9	60.3–83.6	12	21.1	10.5–31.6	0	0.0	––	57	100
	Total	70	60.9	51.9–69.8	33	28.7	20.4–37.0	5	4.3	0.6–8.1	115	100

Knee	Male	76	34.5	28.3–40.8	107	48.6	42.0–55.2	19	8.6	4.9–12.3	220	100
	Female	105	53.3	46.3–60.3	63	32.0	25.5–38.5	19	9.6	5.5–13.8	197	100
	Total	181	43.4	38.6–48.2	170	40.8	36.1–45.5	38	9.1	6.4–11.9	417	100

Lower leg	Male	79	66.9	58.5–75.4	19	16.1	9.5–22.7	15	12.7	6.7–18.7	118	100
	Female	85	74.6	66.6–82.6	8	7.0	2.3–11.7	18	15.8	9.1–22.5	114	100
	Total	164	70.7	64.8–76.5	27	11.6	7.5–15.8	33	14.2	9.7–18.7	232	100

Ankle	Male	131	49.6	43.6–55.7	114	43.2	37.2–49.2	5	1.9	0.2–3.5	264	100
	Female	137	67.8	61.4–74.3	46	22.8	17.0–28.6	9	1.9	1.6–7.3	202	100
	Total	268	57.5	53.0–62.0	160	34.3	30.0–38.6	14	3.0	1.5–4.6	466	100

Foot/toes	Male	32	54.2	41.5–66.9	18	30.5	18.8–42.3	2	3.4	0.0–8.0	59	100
	Female	30	68.2	54.4–81.9	9	20.5	8.5–32.4	3	6.8	0.0–14.3	44	100
	Total	62	60.2	50.7–69.6	27	26.2	17.7–34.7	5	4.9	0.7–9.0	103	100

Other	Male	103	51.2	44.3–58.2	63	31.3	24.9–37.8	10	5.0	2.0–8.0	201	100
	Female	122	68.2	61.3–75.0	28	15.6	10.3–21.0	12	6.7	3.0–10.4	179	100
	Total	225	59.2	61.3–75.0	91	23.9	19.7–28.2	22	5.8	3.4–8.1	380	100

Total	Male	551	48.2	45.4–51.1	405	35	32.7–38.2	76	6.7	5.2–8.1	1142	100
	Female	638	64.6	61.7–67.6	195	19.8	17.3–22.2	80	8.1	6.4–9.8	987	100
	Total	1189	55.8	53.7–58.0	600	28.2	26.3–30.1	156	7.3	6.2–8.4	2129	100

## Data Availability

The data used to support the findings of this study were supplied under license and so cannot be made freely available.

## Supplementary Materials

Supplementary Materials Supplementary file 1: baseline demographics of included students and amounts of registered injuries per curriculum year. Supplementary file 2: injury prevalence per injury location by injury types and sex.

## References

[1] Beijsterveldt V. A., Richardson A., Clarsen B., Stubbe J. H. (2017). Sports Injuries and Illnesses in First-Year Physical Education Teacher Education Students. *BMJ open sport & exercise medicine*.

[2] Bliekendaal S., Goossens L., Stubbe J. H. (2017). Incidence and Risk Factors of Injuries and Their Impact on Academic success: A Prospective Study in PETE Students. *Scand J Med Sci Sports*.

[3] Ehrendorfer S. (1998). Suvey of sports injuries in physical education students participating in 13 sports. *Wiener Klinische Wochenschrift*.

[4] Goossens L., Verrelst R., Cardon G., De Clercq D. (2014). Sports injuries in physical education teacher education students. *Scandinavian Journal of Medicine & Science in Sports*.

[5] Mukherjee S. (2014). Sports injuries in university physical education teacher education students A prospective epidemiological investigation. *J Sports Medicine*.

[6] Twellaar M., Verstappen F. T. J., Huson A. (1996). Is prevention of sports injuries a realistic goal? A four-year prospective investigation of sports injuries among physical education students. *The American Journal of Sports Medicine*.

[7] Drew M. K., Raysmith B. P., Charlton P. C. (2017). Injuries impair the chance of successful performance by sportspeople: a systematic review. *British Journal of Sports Medicine*.

[8] Gouttebarge V., Inklaar H., Frings-Dresen M. H. (2014). Risk and consequences of osteoarthritis after a professional football career: a systematic review of the recent literature. *The Journal of Sports Medicine and Physical Fitness*.

[9] Gribble P. A., Bleakley C. M., Caulfield B. M. (2016). Evidence review for the 2016 International Ankle Consortium consensus statement on the prevalence, impact and long-term consequences of lateral ankle sprains. *British Journal of Sports Medicine*.

[10] Richmond S. A., Fukuchi R. K., Ezzat A., Schneider K., Schneider G., Emery C. A. (2013). Are joint injury, sport activity, physical activity, obesity, or occupational activities predictors for osteoarthritis? A systematic review. *Journal of Orthopaedic & Sports Physical Therapy*.

[11] Goossens L., Cardon G., Witvrouw E., Steyaert A., De Clercq D. (2015). A multifactorial injury prevention intervention reduces injury incidence in Physical Education Teacher Education students. *European Journal of Sport Science*.

[12] Barendrecht M., Barten C. C., Smits-Engelsman B. C. M., Mechelen W., Verhagen E. A. L. M. (2021). A retrospective analysis of injury risk in physical education teacher education students between 2000-2014. *Translational Sports Medicine*.

[13] van Mechelen W., Hlobil H., Kemper H. C. (1992). Incidence, severity, aetiology and prevention of sports injuries. A review of concepts. *Sports Medicine*.

[14] Bliekendaal S., Moen M., Fokker Y., Stubbe J. H., Twisk J., Verhagen E. (2018). Incidence and risk factors of medial tibial stress syndrome: a prospective study in Physical Education Teacher Education students. *BMJ Open Sport & Exercise Medicine*.

[15] Snowden J. M., Klebanoff M. A. (2022). Accurate identification of cohort study designs in perinatal research: a practical guide. *American Journal of Obstetrics and Gynecology*.

[16] Regulation E. U (2016). 679 OF THE EUROPEAN PARLIAMENT AND OF THE COUNCIL of 27 April 2016 on the protection of natural persons with regard to the processing of personal data and on the free movement of such data, and repealing Directive 95/46/EC (General Data Protection Regulation). *Official Journal of the European Union*.

[17] Junge A., Engebretsen L., Alonso J. M. (2008). Injury surveillance in multi-sport events: the International Olympic Committee approach. *British Journal of Sports Medicine*.

[18] Fuller C. W., Ekstrand J., Junge A. (2006). Consensus statement on injury definitions and data collection procedures in studies of football (soccer) injuries. *British Journal of Sports Medicine*.

[19] Barendrecht M., Tak I., Barten C., Verhagen E. (2022). Contribution of sex, sports and activity types and curriculum load distribution to intracurricular injury risk in physical education teacher education: a cohort study. *BMJ Open Sport & Exercise Medicine*.

[20] Jones B. H., Hauschild V. D. (2015). Physical training, fitness, and injuries: lessons learned from military studies. *The Journal of Strength & Conditioning Research*.

[21] Korbee S., Gerritsen H. (2017). *Lidmaatschappen en Sportdeelname NOC^∗^NSF over*.

[22] Bliekendaal S., Barendrecht M., Stubbe J., Verhagen E. (2021). Mechanisms of sport-related injuries in physical education teacher education students: a descriptive analysis of 896 injuries. *Translational Sports Medicine*.

[23] Goossens L., De Ridder R., Cardon G., Witvrouw E., Verrelst R., De Clercq D. (2019). Injury prevention in physical education teacher education students: lessons from sports. A systematic review. *European Physical Education Review*.

[24] Bellows R., Wong C. K. (2018). The effect of bracing and balance training on ankle sprain incidence among athletes: a systematic review with meta-analysis. *International Journal of Sports Physical Therapy*.

[25] Donnell-Fink L. A., Klara K., Collins J. E. (2015). Effectiveness of knee injury and anterior cruciate ligament tear prevention programs: a meta-analysis. *PLoS One*.

[26] Van Dyk N., Behan F. P., Whiteley R. (2019). Including the Nordic hamstring exercise in injury prevention programmes halves the rate of hamstring injuries: a systematic review and meta-analysis of 8459 athletes. *British Journal of Sports Medicine*.

[27] Bengtsson H., Ekstrand J., Hagglund M. (2013). Muscle injury rates in professional football increase with fixture congestion: an 11-yearfollow-up of the UEFA Champions League injury study. *British Journal of Sports Medicine*.

[28] Windt J., Gabbett T. J. (2017). How do training and competition workloads relate to injury? The workload-injury aetiology model. *British Journal of Sports Medicine*.

[29] ter Stege M. H. P., Dallinga J. M., Benjaminse A., Lemmink K. A. P. M. (2014). Effect of interventions on potential, modifiable risk factors for knee injury in team ball sports: a systematic review. *Sports Medicine*.

[30] Myer G. D., Ford K. R., Di Stasi S. L., Foss K. D. B., Micheli L. J., Hewett T. E. (2015). High knee abduction moments are common risk factors for patellofemoral pain (PFP) and anterior cruciate ligament (ACL) injury in girls: is PFP itself a predictor for subsequent ACL injury?. *British Journal of Sports Medicine*.

[31] Meeuwisse W. H., Tyreman H., Hagel B., Emery C. (2007). A dynamic model of etiology in sport injury: the recursive nature of risk and causation. *Clinical Journal of Sport Medicine*.

