# Supplementary material for: Injuries in Physical Education Teacher Students: Differences between Sex, Curriculum Year, Setting, and Sports
Source: Transl Sports Med. 2023 Jan 5;2023:8643402. doi: 10.1155/2023/8643402 (PMC11023724; doi:10.1155/2023/8643402)

Injuries in physical education teacher students - differences between sex curriculum year setting and sports activity - supplementary file 1:

Baseline demographics of included students and amounts of registered injuries per curriculum year.

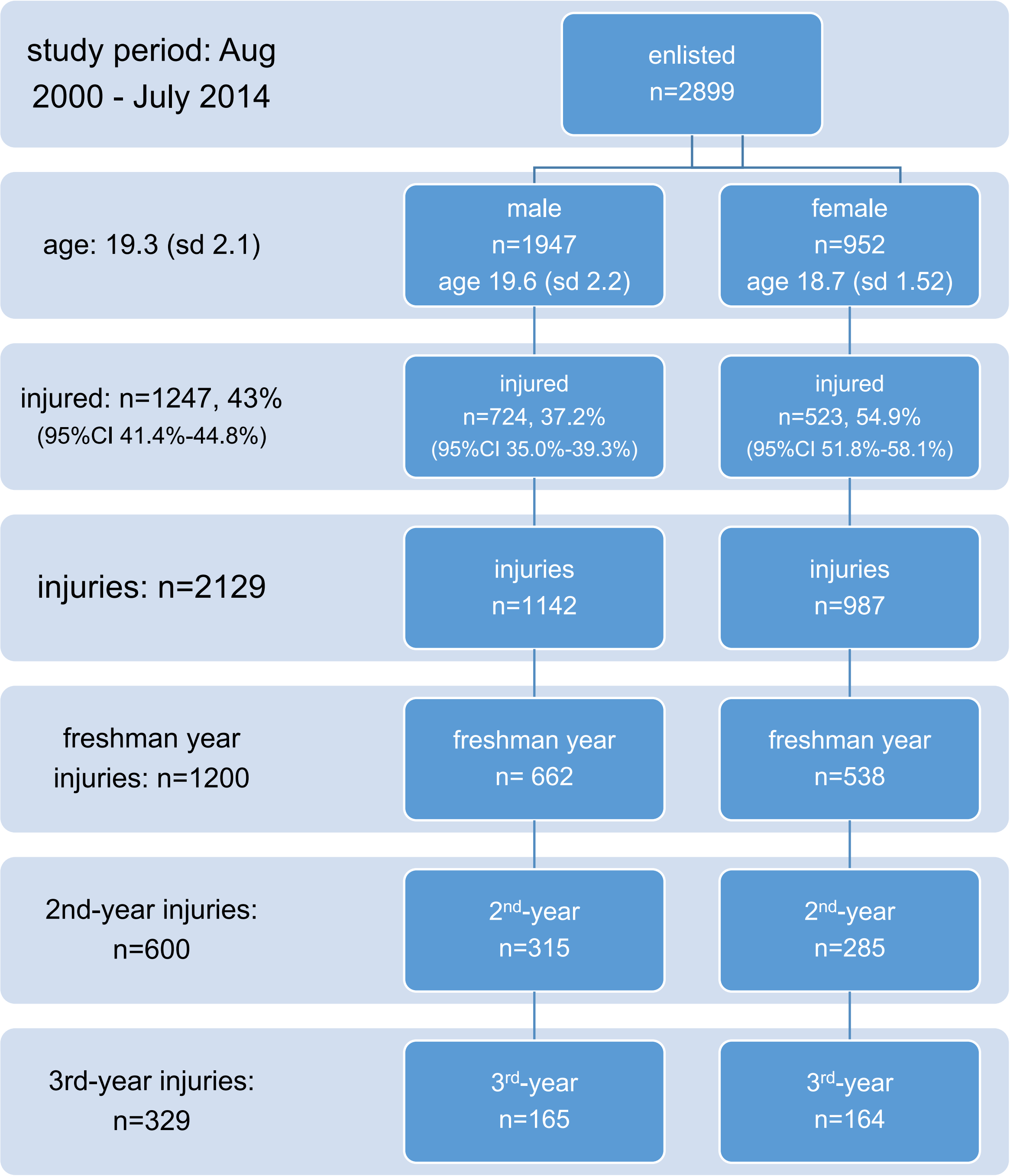

Supplement: Supplementary Materials — Supplementary file 1: baseline demographics of included students and amounts of registered injuries per curriculum year. Supplementary file 2: injury prevalence per injury location by injury types and sex. [file 8643402.f1.zip › Injuries in physical education teacher students - differences between sex curriculum year setting and sports activity supplementary file 1.pdf]
