# Supplementary material for: Injuries in Physical Education Teacher Students: Differences between Sex, Curriculum Year, Setting, and Sports
Source: Transl Sports Med. 2023 Jan 5;2023:8643402. doi: 10.1155/2023/8643402 (PMC11023724; doi:10.1155/2023/8643402)
Supplement: Supplementary Materials — Supplementary file 1: baseline demographics of included students and amounts of registered injuries per curriculum year. Supplementary file 2: injury prevalence per injury location by injury types and sex. [file 8643402.f1.zip › Injuries in physical education teacher students - differences between sex curriculum year setting and sports activity supplementary file 2.pdf]

Injuries in physical education teacher students - differences between sex curriculum year setting and sports activity supplementary file 2:

Injury prevalence per injury location by injury types and sex with 95% confidence intervals.

Injury location		Injury type																
		Fractures and bone stress overall			Joint and ligament overall			Muscle and tendon overall			Haematoma/ contusion/bruise			Other/unknown			Total	
		n	% within Injury location	95%CI	n	% within Injury location	95%CI	n	% within Injury location	95%CI	n	% within Injury location	95%CI	n	% within Injury location	95%CI	n	% within Injury location
**Head and neck: total**	male	2	8,3%	0,0% -19,4%	6	25,0%	7,7% -42,3%	7	29,2%	11,0% -47,4%	4	16,7%	1,8% -31,6%	8	33,3%	14,5% -52,2%	24	100,0%
	female	1	4,0%	0,0% -11,7%	8	32,0%	13,7% -50,3%	5	20,0%	4,3% -35,7%	4	16,0%	1,6% -30,4%	11	44,0%	24,5% -63,5%	25	100,0%
	total	3	6,1%	0,0% -12,8%	14	28,6%	15,9% -41,2%	12	24,5%	12,4% -36,5%	8	16,3%	6,0% -26,7%	19	38,8%	25,1% -52,4%	49	100,0%
Head/face	male	2	22,2%	0,0% -49,4%	1	11,1%	0,0% -31,6%	0	0,0%	0,0% -0,0%	2	22,2%	0,0% -49,4%	7	77,8%	50,6% -104,9%	9	100,0%
	female	1	11,1%	0,0% -31,6%	0	0,0%	0,0% -0,0%	0	0,0%	0,0% -0,0%	3	33,3%	2,5% -64,1%	8	88,9%	68,4% -109,4%	9	100,0%
	total	3	16,7%	0,0% -33,9%	1	5,6%	0,0% -16,1%	0	0,0%	0,0% -0,0%	5	27,8%	7,1% -48,5%	15	83,3%	66,1% -100,6%	18	100,0%
Neck/cervical spine	male	0	0,0%	0,0% -0,0%	5	33,3%	9,5% -57,2%	7	46,7%	21,4% -71,9%	2	13,3%	0,0% -30,5%	1	6,7%	0,0% -19,3%	15	100,0%
	female	0	0,0%	0,0% -0,0%	8	53,3%	28,1% -78,6%	5	33,3%	9,5% -57,2%	1	6,7%	0,0% -19,3%	2	13,3%	0,0% -30,5%	15	100,0%
	total	0	0,0%	0,0% -0,0%	13	43,3%	25,6% -61,1%	12	40,0%	22,5% -57,5%	3	10,0%	0,0% -20,7%	3	10,0%	0,0% -20,7%	30	100,0%
**Upper limbs: total**	male	35	14,4%	10,0% -18,8%	95	39,1%	33,0% -45,2%	70	28,8%	23,1% -34,5%	14	5,8%	2,8% -8,7%	29	11,9%	7,9% -16,0%	243	100,0%
	female	17	8,9%	4,9% -12,9%	82	42,9%	35,9% -50,0%	46	24,1%	18,0% -30,1%	23	12,0%	7,4% -16,7%	24	12,6%	7,9% -17,3%	191	100,0%
	total	52	12,0%	8,9% -15,0%	177	40,8%	36,2% -45,4%	116	26,7%	22,6% -30,9%	37	8,5%	5,9% -11,2%	53	12,2%	9,1% -15,3%	434	100,0%
Shoulder/ clavicular	male	9	6,9%	2,6% -11,3%	45	34,6%	26,4% -42,8%	57	43,8%	35,3% -52,4%	5	3,8%	0,5% -7,2%	14	10,8%	5,4% -16,1%	130	100,0%
	female	0	0,0%	0,0% -0,0%	37	44,0%	33,4% -54,7%	30	35,7%	25,5% -46,0%	6	7,1%	1,6% -12,7%	11	13,1%	5,9% -20,3%	84	100,0%
	total	9	4,2%	1,5% -6,9%	82	38,3%	31,8% -44,8%	87	40,7%	34,1% -47,2%	11	5,1%	2,2% -8,1%	25	11,7%	7,4% -16,0%	214	100,0%
Upper arm	male	0	0,0%	0,0% -0,0%	0	0,0%	0,0% -0,0%	2	100,0%	100,0% -100,0%	0	0,0%	0,0% -0,0%	0	0,0%	0,0% -0,0%	2	100,0%
	female	0	0,0%	0,0% -0,0%	0	0,0%	0,0% -0,0%	1	50,0%	0,0% -119,3%	1	50,0%	0,0% -119,3%	0	0,0%	0,0% -0,0%	2	100,0%
	total	0	0,0%	0,0% -0,0%	0	0,0%	0,0% -0,0%	3	75,0%	32,6% -117,4%	1	25,0%	0,0% -67,4%	0	0,0%	0,0% -0,0%	4	100,0%
Elbow	male	2	11,1%	0,0% -25,6%	8	44,4%	21,5% -67,4%	5	27,8%	7,1% -48,5%	1	5,6%	0,0% -16,1%	2	11,1%	0,0% -25,6%	18	100,0%
	female	2	10,5%	0,0% -24,3%	6	31,6%	10,7% -52,5%	6	31,6%	10,7% -52,5%	4	21,1%	2,7% -39,4%	2	10,5%	0,0% -24,3%	19	100,0%
	total	4	10,8%	0,8% -20,8%	14	37,8%	22,2% -53,5%	11	29,7%	15,0% -44,5%	5	13,5%	2,5% -24,5%	4	10,8%	0,8% -20,8%	37	100,0%
Forearm	male	1	16,7%	0,0% -46,5%	0	0,0%	0,0% -0,0%	3	50,0%	10,0% -90,0%	1	16,7%	0,0% -46,5%	1	16,7%	0,0% -46,5%	6	100,0%
	female	1	16,7%	0,0% -46,5%	1	16,7%	0,0% -46,5%	3	50,0%	10,0% -90,0%	1	16,7%	0,0% -46,5%	0	0,0%	0,0% -0,0%	6	100,0%
	total	2	16,7%	0,0% -37,8%	1	8,3%	0,0% -24,0%	6	50,0%	21,7% -78,3%	2	16,7%	0,0% -37,8%	1	8,3%	0,0% -24,0%	12	100,0%
Wrist	male	13	30,2%	16,5% -44,0%	16	37,2%	22,8% -51,7%	0	0,0%	0,0% -0,0%	4	9,3%	0,6% -18,0%	10	23,3%	10,6% -35,9%	43	100,0%
	female	6	18,2%	5,0% -31,3%	11	33,3%	17,2% -49,4%	2	6,1%	0,0% -14,2%	5	15,2%	2,9% -27,4%	9	27,3%	12,1% -42,5%	33	100,0%
	total	19	25,0%	15,3% -34,7%	27	35,5%	24,8% -46,3%	2	2,6%	0,0% -6,2%	9	11,8%	4,6% -19,1%	19	25,0%	15,3% -34,7%	76	100,0%
Hand/finger/thumb	male	10	22,7%	10,3% -35,1%	26	59,1%	44,6% -73,6%	3	6,8%	0,0% -14,3%	3	6,8%	0,0% -14,3%	2	4,5%	0,0% -10,7%	44	100,0%
	female	8	17,4%	6,4% -28,3%	27	58,7%	44,5% -72,9%	4	8,7%	0,6% -16,8%	6	13,0%	3,3% -22,8%	1	2,2%	0,0% -6,4%	46	100,0%
	total	18	20,0%	11,7% -28,3%	53	58,9%	48,7% -69,1%	7	7,8%	2,2% -13,3%	9	10,0%	3,8% -16,2%	3	3,3%	0,0% -7,0%	90	100,0%
**Trunk total**	male	1	1,2%	0,0% -3,5%	35	41,7%	31,1% -52,2%	17	20,2%	11,6% -28,8%	13	15,5%	7,7% -23,2%	18	21,4%	12,7% -30,2%	84	100,0%
	female	2	2,0%	0,0% -4,8%	37	37,4%	27,8% -46,9%	23	23,2%	14,9% -31,6%	9	9,1%	3,4% -14,8%	28	28,3%	19,4% -37,2%	99	100,0%
	total	3	1,6%	0,0% -3,5%	72	39,3%	32,3% -46,4%	40	21,9%	15,9% -27,8%	22	12,0%	7,3% -16,7%	46	25,1%	18,9% -31,4%	183	100,0%
Sternum/ribs	male	1	9,1%	0,0% -26,1%	0	0,0%	0,0% -0,0%	1	9,1%	0,0% -26,1%	9	81,8%	59,0% -104,6%	0	0,0%	0,0% -0,0%	11	100,0%
	female	1	25,0%	0,0% -67,4%	0	0,0%	0,0% -0,0%	2	50,0%	1,0% -99,0%	1	25,0%	0,0% -67,4%	0	0,0%	0,0% -0,0%	4	100,0%
	total	2	13,3%	0,0% -30,5%	0	0,0%	0,0% -0,0%	3	20,0%	0,0% -40,2%	10	66,7%	42,8% -90,5%	0	0,0%	0,0% -0,0%	15	100,0%
Abdomen	male	0	0,0%	0,0% -0,0%	0	0,0%	0,0% -0,0%	1	50,0%	0,0% -119,3%	1	50,0%	0,0% -119,3%	0	0,0%	0,0% -0,0%	2	100,0%
	female	0	0,0%	0,0% -0,0%	0	0,0%	0,0% -0,0%	0	0,0%	0,0% -0,0%	0	0,0%	0,0% -0,0%	1	100,0%	100,0% -100,0%	1	100,0%
	total	0	0,0%	0,0% -0,0%	0	0,0%	0,0% -0,0%	1	33,3%	0,0% -86,7%	1	33,3%	0,0% -86,7%	1	33,3%	0,0% -86,7%	3	100,0%
Back (upper and lower)/ pelvis/sacrum	male	0	0,0%	0,0% -0,0%	35	49,3%	37,7% -60,9%	15	21,1%	11,6% -30,6%	3	4,2%	0,0% -8,9%	18	25,4%	15,2% -35,5%	71	100,0%
	female	1	1,1%	0,0% -3,1%	37	39,4%	29,5% -49,2%	21	22,3%	13,9% -30,8%	8	8,5%	2,9% -14,2%	27	28,7%	19,6% -37,9%	94	100,0%
	total	1	0,6%	0,0% -1,8%	72	43,6%	36,1% -51,2%	36	21,8%	15,5% -28,1%	11	6,7%	2,9% -10,5%	45	27,3%	20,5% -34,1%	165	100,0%
**Lower Limbs: total**	male	100	13,0%	10,6% -15,3%	398	51,6%	48,1% -55,1%	167	21,7%	18,8% -24,6%	39	5,1%	3,5% -6,6%	68	8,8%	6,8% -10,8%	771	100,0%
	female	102	15,6%	12,8% -18,3%	314	47,9%	44,1% -51,8%	160	24,4%	21,1% -27,7%	20	3,1%	1,7% -4,4%	61	9,3%	7,1% -11,5%	655	100,0%
	total	202	14,2%	12,4% -16,0%	712	49,9%	47,3% -52,5%	327	22,9%	20,7% -25,1%	59	4,1%	3,1% -5,2%	129	9,0%	7,6% -10,5%	1426	100,0%
Hip/groin	male	0	0,0%	0,0% -0,0%	0	0,0%	0,0% -0,0%	30	83,3%	71,2% -95,5%	0	0,0%	0,0% -0,0%	6	16,7%	4,5% -28,8%	36	100,0%
	female	0	0,0%	0,0% -0,0%	0	0,0%	0,0% -0,0%	19	73,1%	56,0% -90,1%	1	3,8%	0,0% -11,2%	6	23,1%	6,9% -39,3%	26	100,0%
	total	0	0,0%	0,0% -0,0%	0	0,0%	0,0% -0,0%	49	79,0%	68,9% -89,2%	1	1,6%	0,0% -4,7%	12	19,4%	9,5% -29,2%	62	100,0%
Thigh	male	0	0,0%	0,0% -0,0%	0	0,0%	0,0% -0,0%	53	91,4%	84,2% -98,6%	5	8,6%	1,4% -15,8%	0	0,0%	0,0% -0,0%	58	100,0%
	female	0	0,0%	0,0% -0,0%	0	0,0%	0,0% -0,0%	52	91,2%	83,9% -98,6%	3	5,3%	0,0% -11,1%	3	5,3%	0,0% -11,1%	57	100,0%
	total	0	0,0%	0,0% -0,0%	0	0,0%	0,0% -0,0%	105	87,0%	80,8% -93,1%	8	7,0%	2,3% -11,6%	3	2,6%	0,0% -5,5%	115	100,0%
Knee	male	2	0,9%	0,0% -2,2%	156	70,9%	64,9% -76,9%	32	14,5%	9,9% -19,2%	8	3,6%	1,2% -6,1%	22	10,0%	6,0% -14,0%	220	100,0%
	female	2	1,0%	0,0% -2,4%	122	61,9%	55,1% -68,7%	34	17,3%	12,0% -22,5%	11	5,6%	2,4% -8,8%	28	14,2%	9,3% -19,1%	197	100,0%
	total	4	1,0%	0,0% -1,9%	278	66,7%	62,1% -71,2%	66	15,8%	12,3% -19,3%	19	4,6%	2,6% -6,6%	50	12,0%	8,9% -15,1%	417	100,0%
Lower leg	male	82	69,5%	61,2% -77,8%	0	0,0%	0,0% -0,0%	24	20,3%	13,1% -27,6%	9	7,6%	2,8% -12,4%	3	2,5%	0,0% -5,4%	118	100,0%
	female	87	76,3%	68,5% -84,1%	1	0,9%	0,0% -2,6%	22	19,3%	12,1% -26,5%	0	0,0%	0,0% -0,0%	4	3,5%	0,1% -6,9%	114	100,0%
	total	169	72,8%	67,1% -78,6%	1	0,4%	0,0% -1,3%	46	19,8%	14,7% -25,0%	9	3,9%	1,4% -6,4%	7	3,0%	0,8% -5,2%	232	100,0%
Achilles tendon	male	0	0,0%	0,0% -0,0%	0	0,0%	0,0% -0,0%	12	100,0%	100,0% -100,0%	0	0,0%	0,0% -0,0%	0	0,0%	0,0% -0,0%	12	100,0%
	female	0	0,0%	0,0% -0,0%	0	0,0%	0,0% -0,0%	12	100,0%	100,0% -100,0%	0	0,0%	0,0% -0,0%	0	0,0%	0,0% -0,0%	12	100,0%
	total	0	0,0%	0,0% -0,0%	0	0,0%	0,0% -0,0%	24	100,0%	100,0% -100,0%	0	0,0%	0,0% -0,0%	0	0,0%	0,0% -0,0%	24	100,0%
Ankle	male	6	2,3%	0,5% -4,1%	235	89,0%	85,2% -92,8%	3	1,1%	0,0% -2,4%	2	0,8%	0,0% -1,8%	18	6,8%	3,8% -9,9%	264	100,0%
	female	3	1,5%	0,0% -3,2%	185	91,6%	87,8% -95,4%	4	2,0%	0,1% -3,9%	0	0,0%	0,0% -0,0%	10	5,0%	2,0% -7,9%	202	100,0%
	total	9	1,9%	0,7% -3,2%	420	90,1%	87,4% -92,8%	7	1,5%	0,4% -2,6%	2	0,4%	0,0% -1,0%	28	6,0%	3,9% -8,2%	466	100,0%
Foot/toe	male	10	16,9%	7,4% -26,5%	7	11,9%	3,6% -20,1%	13	22,0%	11,5% -32,6%	13	22,0%	11,5% -32,6%	17	28,8%	17,3% -40,4%	59	100,0%
	female	10	22,7%	10,3% -35,1%	5	11,4%	2,0% -20,7%	16	36,4%	22,1% -50,6%	5	11,4%	2,0% -20,7%	9	20,5%	8,5% -32,4%	44	100,0%
	total	20	19,4%	11,8% -27,1%	12	11,7%	5,5% -17,8%	29	28,2%	19,5% -36,8%	18	17,5%	10,1% -24,8%	26	25,2%	16,9% -33,6%	103	100,0%
**Unknown**	male	0	0,0%	0,0% -0,0%	1	5,0%	0,0% -14,6%	0	0,0%	0,0% -0,0%	2	10,0%	0,0% -23,1%	17	85,0%	69,4% -100,6%	20	100,0%
	female	0	0,0%	0,0% -0,0%	0	0,0%	0,0% -0,0%	3	17,6%	0,0% -35,8%	0	0,0%	0,0% -0,0%	14	82,4%	64,2% -100,5%	17	100,0%
	total	0	0,0%	0,0% -0,0%	1	2,7%	0,0% -7,9%	3	8,1%	0,0% -16,9%	2	5,4%	0,0% -12,7%	31	83,8%	71,9% -95,7%	37	100,0%
**Total**	male	138	12,1%	10,2% -14,0%	535	46,8%	44,0% -49,7%	261	22,9%	20,4% -25,3%	72	6,3%	4,9% -7,7%	140	12,3%	10,4% -14,2%	1142	100,0%
	female	122	12,4%	10,3% -14,4%	441	44,7%	41,6% -47,8%	237	24,0%	21,3% -26,7%	56	5,7%	4,2% -7,1%	138	14,0%	11,8% -16,1%	987	100,0%
	total	260	12,2%	10,8% -13,6%	976	45,8%	43,7% -48,0%	498	23,4%	21,6% -25,2%	128	6,0%	5,0% -7,0%	278	13,1%	11,6% -14,5%	2129	100,0%

Supplementary table1: Injury prevalence per injury location by injury types and sex with 95% confidence intervals. Proportions were calculated in percentages from the total per sex within each injury location.
